# Manual versus Automated moNitoring Accuracy of GlucosE II (MANAGE II)

**DOI:** 10.1186/s13054-016-1547-3

**Published:** 2016-11-25

**Authors:** Cláudia Righy Shinotsuka, Alexandre Brasseur, David Fagnoul, Timothy So, Jean-Louis Vincent, Jean-Charles Preiser

**Affiliations:** 1 Department of Intensive Care, Erasme University Hospital, Université libre de Bruxelles, 808 route de Lennik, Brussels, B-1070 Belgium; 2 Optiscan Corporation, Hayward, CA USA

## Abstract

**Background:**

Intravascular continuous glucose monitoring (CGM) may facilitate glycemic control in the intensive care unit (ICU). We compared the accuracy of a CGM device (OptiScanner®) with a standard reference method.

**Methods:**

Adult patients who had blood glucose (BG) levels >150 mg/dl and required insertion of an arterial and central venous catheter were included. The OptiScanner® was inserted into a multiple-lumen central venous catheter. Patients were treated using a dynamic-scale insulin algorithm to achieve BG values between 80 and 150 mg/dl. The BG values measured by the OptiScanner® were plotted against BG values measured using a reference analyzer. The correlation between the BG values measured using the two methods and the clinical relevance of any differences were assessed using the coefficient of determination (*r* ^2^) and the Clarke error grid, respectively; bias was assessed by the mean absolute relative difference (MARD). Three different standards of glucose monitoring were used to assess accuracy. Glycemic control was assessed using the time in range (TIR). Six indices of glycemic variability were calculated.

**Results:**

The analysis included 929 paired samples from 88 patients, monitored for a total of 2584 hours. Reference BG values ranged between 60 and 484 mg/dl. The *r* ^2^ value was 0.89. The percentage of BG values within zones A and B of the Clarke error grid was 99.9%; the MARD was 7.7%. Using the ISO 15197 standard and Food and Drug Administration and consensus standards, respectively, 80.4% of measurements were within 15 mg/dl and 88.2% within 15% of reference values, 40% of measurements were within 7 mg/dl and 72.5% within 10% of reference values, and 65.2% of measurements were within 10 mg/dl and 82.7% within 12.5% of reference values. The TIR was slightly lower with the OptiScanner® than with the reference method. The J-index, standard deviation and maximal glucose change were the indices of glycemic variability least affected by the measurement device.

**Conclusions:**

Based on the MARD, the performance of the OptiScanner® is adequate for use in ICU patients. Because recent standards for accuracy were not met, the OptiScanner® should not be used as a sole monitor. The assessment of glycemic variability is influenced by the time interval between BG determinations.

**Trial registration:**

Clinicaltrials.gov NCT01720381. Registered 31 October 2012.

## Background

The topic of glycemic control in intensive care unit (ICU) patients fuels vivid debate. Indeed, some experts advocate tight glycemic control (TGC) [1] while others support a “no-touch” approach in cases of stress hyperglycemia [2]. Both views are supported by plausible physiological rationales, although clinical data are highly controversial [3]. Although retrospective data collected in various settings have reported that hyperglycemia, hypoglycemia and glycemic variability are independent and synergistic risk factors for increased mortality and morbidity, prospective studies comparing TGC with liberal glucose control have given divergent results [3, 4]. The survival benefit associated with TGC using intensive insulin therapy reported in the initial landmark study by Van den Berghe et al. [5] was not confirmed in later multicenter trials [4, 6–8]. Indeed, the 90-day mortality rate observed in the largest of these trials [6] was higher in the TGC group than in the control group. These major differences among studies are only partially understood and could be related to various factors, including differences in the ability to achieve the predefined blood glucose target, and an increased incidence of hypoglycemia and higher glycemic variability induced by the intensive insulin therapy.

Achieving TGC or an intermediate level of glycemic control necessitates a complex multiple-step therapeutic modality [9], implying that future interventional studies will need improved treatment strategies [10–12]. Among recent technological improvements, continuous or near-continuous intravascular glucose monitoring (CGM) systems represent a promising means of potentially facilitating glucose control and decreasing the nursing workload associated with TGC. However, validation of available CGM systems in real-world conditions is a necessary step before the large-scale dissemination of these devices. In terms of glucose metrics, replacement of intermittent readings by CGM-derived data will also require careful reassessment, as was the case for patients with type I diabetes, in whom interstitial CGM systems are now widely used.

The aims of the present study were: to validate and assess the accuracy of the latest version of a mid-infrared spectroscopy-based CGM system (OptiScanner®; Optiscan Biomedical Corporation, Hayward, CA, USA) designed for use in the ICU, against a reference blood gas analyzer method; and to compare glycemic variability indices calculated from intermittent readings and CGM values.

## Methods

### Study design

This study was performed in the 35-bed mixed Department of Intensive Care of Erasme University Hospital in Brussels, Belgium. It was an investigator-initiated study of critically ill patients who required blood glucose control with intravenous insulin, according to local policy that targets a blood glucose level between 80 and 150 mg/dl. The study was approved by the hospital ethics committee and patients (or a legal representative) provided written informed consent before any study-related activity. The study was registered at Clinicaltrials.gov (NCT 01720381).

### Study population

Patients were eligible for inclusion if they were aged ≥ 18 years, had an expected ICU stay ≥ 3 days at the time of enrollment, had an Acute Physiology and Chronic Health Evaluation (APACHE) II [13] score ≥ 10 within the first 24 hours of ICU admission, had blood glucose measured at the time of ICU admission > 150 mg/dl and required insertion of an arterial catheter and a central venous catheter. Included patients were not allowed to participate in any other concurrent investigational interventional study. The only exclusion criterion was pregnancy.

### Glycemic control in the ICU

A computerized dynamic-scale insulin protocol adapted from Meynaar et al. [14] and operated by nurses is used to achieve glycemic control in our ICU. The protocol takes into account the previous blood glucose value, the current blood glucose value, the current insulin infusion rate and the carbohydrate intake. After providing this information to the computer, the protocol recommends therapeutic changes (insulin bolus and/or infusion rate or amount of glucose if hypoglycemic) and the time interval for the next blood glucose check (minimum 6 hourly). Severe hyperglycemia was defined as blood glucose > 180 mg/dl, while severe and mild hypoglycemia were respectively defined as blood glucose values < 40 and 41–70 mg/dl.

### Study procedures

Once informed consent had been obtained, a radial or femoral arterial catheter and a 4-lumen jugular or subclavian central venous catheter (20 cm, 8.5 F; Arrow Int., Morrisville, NC, USA) were inserted. The OptiScanner® was connected to a dedicated proximal lumen of the central venous catheter.

The OptiScanner® withdraws 3 ml of blood every 15 minutes. For each measurement, a sample of 0.1 ml is heparinized and spun in a microcentrifuge, and the plasma sample is then analyzed by a mid-infrared spectrometer. After obtaining the absorption spectrum, the system determines the blood glucose value. The remaining 2.9 ml of blood is returned to the subject with a small amount of saline. There is no heparin in the blood returned to the patient.

Arterial blood draws were taken up to 12 times per day, with at least 1 hour between samples. Each comparative blood sample was collected at the same time as the OptiScanner® was drawing blood from the central venous catheter. The blood glucose in the arterial comparative blood draw was measured using a YSI 2300 STAT Plus analyzer (Yellow Spring Instruments, Yellow Spring, OH¸USA). Two readings were obtained on the same YSI for each sample. Caregivers were blinded to the OptiScanner® results.

Patients with only one paired sample were excluded from the analysis.

### Recorded data

Demographic data (sex, age, ethnicity, history of diabetes as recorded in the medical file), type of admission (medical, surgical, trauma), APACHE II score, ICU and hospital mortality as well as ICU and hospital lengths of stay were recorded.

The “down-time” of the OptiScanner® was calculated as the time without a blood glucose determination, related to monitor, cartridge or catheter issues, when a display of blood glucose values was expected. Monitor-related issues were reported as the no-read rate.

### Statistical analysis

Individual traces were drawn for each patient. OptiScanner® readings were regressed against paired reference values using an unweighted least-squares method. The coefficient of determination (*r* ^2^) was used to assess the linearity over the study measurement range and also as a predictor of glucose prediction error. Bias was assessed using the mean absolute relative difference (MARD) and a Bland–Altman plot was drawn to display the mean difference between OptiScanner® and reference device measurements and the corresponding limits of agreement (mean bias ± 2.0 × standard deviation (SD) of the bias).

The dispersion of blood glucose values was assessed using the population coefficient of variation:

$$ \mathrm{P}\mathrm{C}\mathrm{V} = \left[1 + \left(1/4N\right)\right] \times \mathrm{C}\mathrm{V}, $$

where *N* is the sample size and CV is the coefficient of variation.

A Clarke error grid (CEG) [15] was used to calculate the potential clinical impact of inaccuracies. A CEG is divided into 10 zones: in the two A zones, the device results are <20% of the reference results or the results are <70 mg/dl and the device is considered clinically accurate; in the two B zones, the device results differ by >20% from the reference results, but the differences are considered clinically acceptable because they have little or no effect on treatment decisions; in the C zones, device results differ by >20% from the reference results, and the differences may lead to unnecessary treatment; in the D zones, potentially severe hypoglycemic or hyperglycemic episodes go undetected; and in the E zones, the devices have opposite results, leading to conflicting treatment decisions.

Two different standards of glucose monitoring dedicated to the use of point-of-care meters in diabetology (ISO 15197 2013 and the Food and Drug Administration (FDA) draft guidance of 2014) and the standard suggested for CGM by a board of experts [10] were used to assess accuracy. The ISO 15197 states that 95% of measurements must be within 15% or 15 mg/dl of the reference value (for reference values >100 mg/dl or <100 mg/dl, respectively). For the FDA determination, 99% of measurements must be within 10% of the reference value (if reference value >70 mg/dl) or within 7 mg/dl (if reference value <70 mg/dl) and 100% of measurements must be within 20% (if reference value >70 mg/dl) or within 15 mg/dl (if reference value <70 mg/dl) of the reference value. Using the consensus standard, 98% of measurements must be within 12.5% or 10 mg/dl of the reference value (if reference value >100 mg/dl or <100 mg/dl, respectively) and 2% of the remaining measurements must be within 20% of the reference value.

To assess whether indices of performance and variability were influenced by the time lapse between readings, the intermittent readings determined by the reference device and CGM values from the OptiScanner® were used to calculate the time in range (TIR; percentage of values within the 80–150 mg/dl target range) and six indices of glycemic variability (SD, mean amplitude of glycemic excursions (MAGE), maximal glucose change (MGC), J-index, glucose variability index (GVI) and glucose lability index (GLI)) [16–18]. Glycemic variability indices took into account all of the values registered by the CGM during the same time interval in which YSI values were obtained. Correlations and agreements among these measures of glycemic variability were obtained using Pearson’s correlation coefficient and Bland–Altman methods, respectively.

Data are summarized using means with SD, medians and interquartile ranges (IQRs), or numbers and percentages. Data were analyzed using IBM® SPSS® Statistics software, version 23 for windows and R software, version 3.2.2 (CRAN project). All reported *p* values are two-sided and *p* < 0.05 was considered to indicate statistical significance.

## Results

Ninety-eight patients were included between July 2012 and April 2014; 10 were excluded from the analysis (because of blood-draw issues related to the central venous catheter (*N* = 3), removal of an arterial line (*N* = 2), death before insertion of the OptiScanner® device (*N* = 2) and a small number of paired values (*N* = 3)), leaving 88 patients for the final analysis. The characteristics of the patients are presented in Table 1; 57 (65%) were male, 52 (59%) were admitted for medical reasons and the median APACHE II score was 19.

**Table 1:** Patient characteristics

Characteristic	Value
Male, number (%)	57 (65%)
Age (years), median (IQR)	63 (49–75)
Ethnicity, number (%)	
Asian	3 (4%)
Black	9 (10%)
Caucasian	75 (85%)
Hispanic	1 (1%)
Type of admission, number (%)	
Medical	52 (59%)
Surgical	27 (31%)
Trauma	9 (10%)
Subjects with history of diabetes, number (%)	14 (16%)
APACHE II score, median (IQR)	19 (14–26)
ICU mortality, number (%)	17 (19%)
Hospital mortality, number (%)	18 (20%)
ICU LOS (days), median (IQR)	4 (2–8)
Hospital LOS (days), median (IQR)	11 (7–24)
Duration of monitoring with OptiScanner® (hours), median (IQR)	24 (20–32)

*IQR* interquartile range, *APACHE* Acute Physiology and Chronic Health Evaluation, *ICU* intensive care unit, *LOS* length of stay

A total of 9369 samples (944 paired samples) were drawn by the CGM device during a monitoring period of 2584 hours. For 15 pairs of samples no reading was obtained from the OptiScanner®, leaving a total of 929 pairs for comparison. The “downtime” for the OptiScanner® was 18.9%, including a no-read rate of 7.3%. Some interferents that had not previously been reported, including gelatins (Geloplasma®), were identified during the study and the concomitant infusion of hydroxyethylstarch (Voluven®) and mannitol also interfered with the OptiScanner®. The interference library, which automatically adjusts the glucose measurement algorithm for interferences, was updated accordingly. After reanalyzing the data using the new interference library, and processing the data backwards, the final “no read” rate was 1.6%, yielding a downtime of 13.8%. The OptiScanner® had to be prematurely disconnected in 12 patients because of device/catheter-related issues, including cartridge malfunction, occlusion due to blood draw issues and air in the line.

Blood glucose data calculated from the intermittent reference readings and from the OptiScanner® are presented in Table 2. No episodes of severe hypoglycemia were identified. Three episodes of mild hypoglycemia were detected by the OptiScanner®, but not by the YSI, following a progressive downslope in three patients. More than 50% of the patients had severe hyperglycemia.

**Table 2:** Glucose metrics

Variable	YSI^a^	OptiScanner®^b^
Number of measurements in the ICU	944	929
Number of measurements per patient, median (IQR)	9 (7–12)	9 (7–12)
Blood glucose level (mg/dl), median (IQR)	140.5 (122–169)	145 (123–169.5)
Blood glucose level (mg/dl), mean ± SD	150.6 ± 44.5	150.5 ± 42.2
Range of glucose (mg/dl)	59.7–484	57–436
Measurements of severe hypoglycemia (<40 mg/dl), number (%)	0	0
Patients with severe hypoglycemia, number (%)	0	0
Measurements of mild hypoglycemia (41–70 mg/dl), number (%)	5 (0.5%)	6 (0.6%)
Patients with mild hypoglycemia, number (%)	4 (5%)	3 (3%)
Measurements of severe hyperglycemia (>180 mg/dl), number (%)	167 (18%)	157 (17%)
Patients with severe hyperglycemia, number (%)	46 (52%)	47 (53%)

OptiScanner® glucose metrics are based on the measurements that have a corresponding value for YSI

^a^YSI 2300 STAT Plus analyzer (Yellow Spring Instruments, Yellow Spring, OH, USA)

^b^Optiscan Biomedical Corporation, Hayward, CA, USA

*ICU* intensive care unit, *IQR* interquartile range, *SD* standard deviation

**Fig. 1:**
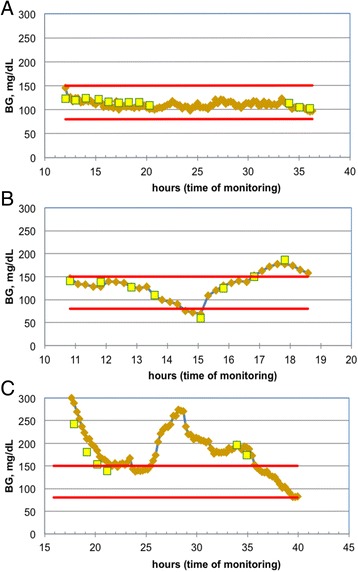
Representative individual traces of blood glucose (*BG*) recordings using the OptiScanner® (*brown lozenges*) and the YSI blood gas analyzer (*yellow squares*). Target range delimited by *red lines*. **a** Well-controlled glycemia with 100% in target range with both methods. **b** Progressive hypoglycemia detected by the OptiScanner® and confirmed later by the blood gas analyzer. **c** Glycemic excursions detected by the OptiScanner® only, with blood gas analyzer-based control satisfactory

Representative traces from individual patients are shown in Fig. 1, showing good control over a relatively prolonged period (Fig. 1a), progressive hypoglycemia detected by the OptiScanner® and confirmed by the reference values (Fig. 1b), and a poorly controlled patient with spikes of hyperglycemia undetected by the reference device (Fig. 1c).

**Fig. 2:**
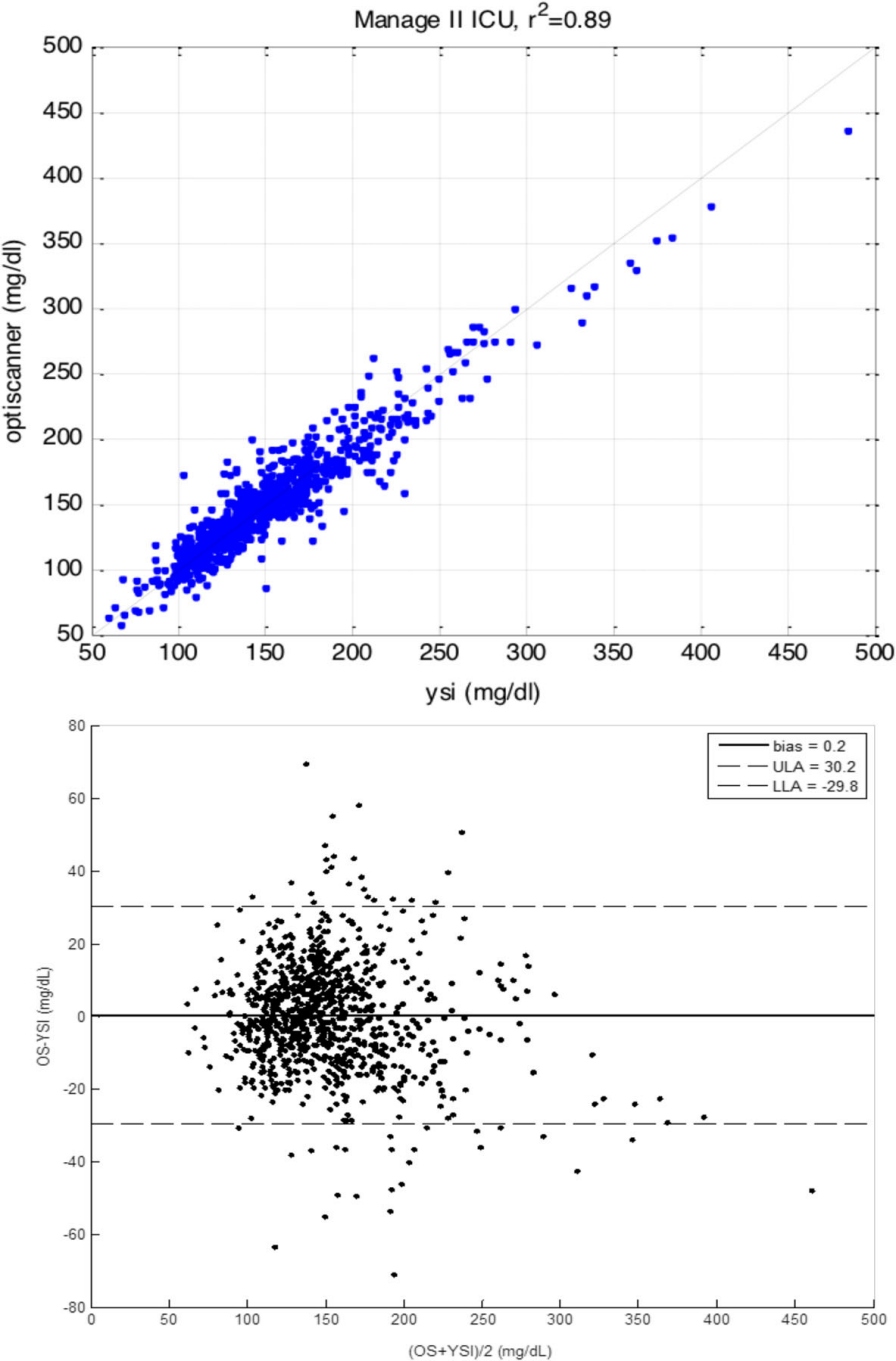
Coefficient of determination (*r* ^2^) and Bland–Altman plot. *Top panel* Correlation between blood glucose measured by the OptiScanner® (*Y* axis) and that measured using the YSI blood gas analyzer (*X* axis) with a statistically significant *p* value. *Lower panel* Bias and limits of agreement between blood glucose measured by the OptiScanner® (*OS*) and by the YSI (Bland–Altman method). *LLA* lower limit of agreement, *ULA* upper limit of agreement

**Fig. 3:**
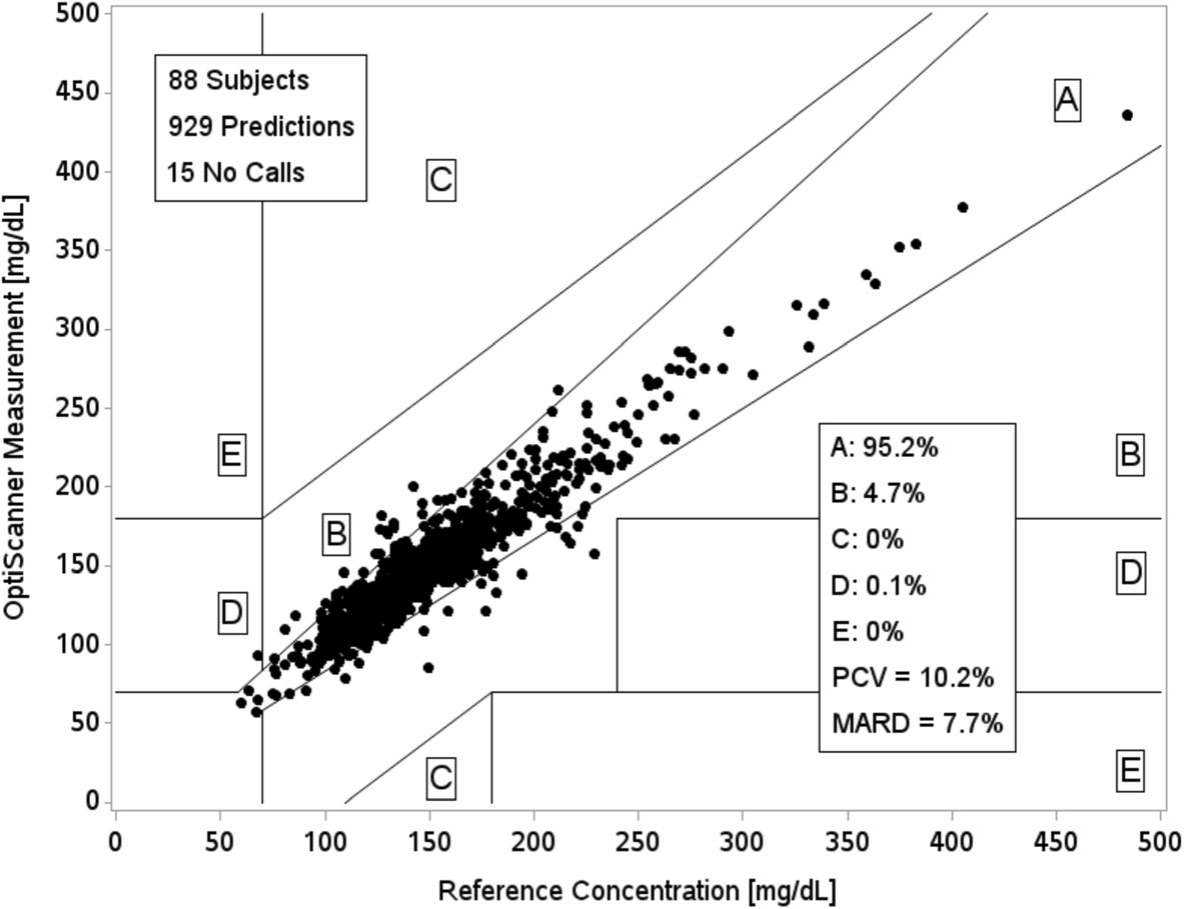
Clark error grid. Paired sample values from the OptiScanner® and the blood gas analyzer (*black dots*). No calls are the number of times that the device was unable to read the blood glucose value (no reads). *PCV* population coefficient of variation, *MARD* mean absolute relative difference

The correlation between the OptiScanner® blood glucose and the reference blood glucose was almost linear (coefficient of determination 0.89; Fig. 2, top panel). The bias and the limits of agreement were satisfactory, as reflected by a MARD of 7.7% (Fig. 3) and as shown on the Bland–Altman plot (Fig. 2, lower panel). The dispersion was low (PCV 10.2%). The CEG (Fig. 3) showed good accuracy of the OptiScanner®, with 95.2% of the results in zone A and 99.9% of the results in zones A + B.

Using the ISO 15197 2013 standard, 80.4% of OptiScanner® measurements were within 15 mg/dl of reference values (for reference values <100 mg/dl) and 88.2% of measurements were within 15% of reference values (for reference values >100 mg/dl). Using the 2014 FDA draft guidance, 40% of measurements were within 7 mg/dl of reference values (for reference values <70 mg/dl) and 72.5% were within 10% of reference values (for reference values >70 mg/dl). Using the 2013 consensus recommendation [10], 65.2% of measurements were within 10 mg/dl of reference values (for reference values <100 mg/dl) and 82.7% of measurements were within 12.5% of reference values (for reference values >100 mg/dl); also, 95.1% of measurements deviated less than 20% from the standard reference value.

The TIR was slightly lower with the OptiScanner® than with the reference method (55.7% and 59.4%, *p* < 0.01). Glucose variability was influenced by the device used for measurement (Table 3). For each GVI, there was a significant correlation (*p* < 0.01) between values calculated from intermittent readings and those calculated by the OptiScanner®. However, correlations were greater for the time-independent indices (i.e., J-index, SD and MGC) than for the time-weighted indices (i.e., GVI and GLI).

**Table 3:** Accuracy and bias (with limits of agreement) of glycemic variability indices obtained from the OptiScanner® and the reference blood gas analyzer

		J-index (mg/dl)^2^	SD (mg/dl)	MGC (mg/dl)	MAGE (mg/dl)	GVI (mg/dl) per hour	GLI (mg/dl) per hour
Accuracy	Correlation (*r*)^a^	0.96	0.94	0.85	0.72	0.52	0.43
	Lower limit	−16.16	−15.35	−45.13	−53.30	−0.95	−967.54
Bias	Mean difference	−1.42	−1.81	−1.01	−3.78	16.54	24.52
	Upper limit	13.32	11.73	43.10	45.73	34.04	1016.59

^a^All values are statistically significant

*SD* standard deviation, *MGC* maximal glucose change, *MAGE* mean amplitude of glycemic excursions, *GVI* glucose variability index, *GLI* glucose lability index

## Discussion

The present study, performed on a heterogeneous population of ICU patients with high disease severity, confirmed the accuracy, as assessed by the MARD, of the mid-infrared spectroscopy-based intravascular CGM device, the OptiScanner®. The device showed good accuracy and a low bias over a period up to 32 hours. These findings are consistent with results published in a former validation study performed with the same device over shorter periods in 71 patients [19], and with the legal requirements of regulatory agencies (CEG zones A + B > 99%). Good correlation between blood glucose values obtained from the OptiScanner® and those obtained using a reference method has also been reported in vivo in an animal study [20], in patients with diabetes [21] and in critically ill patients [19, 22, 23]. CEG analysis and other techniques, such as the Bland–Altman method, have been used to assess discrete point-of-care measurement technologies for a considerable period of time. However, these techniques do not capture the value of trends in data and may understate the possible benefit of a continuous or near-continuous glucose monitor, with the potential for timely alarms. MARD < 10%, however, supports the accuracy of the OptiScanner®, implying a low risk of clinical errors and early detection of hypoglycemia [24, 25].

The OptiScanner® did not meet the requirements for the ISO 15197 2013 standard, the FDA guidance or the consensus recommendation. However, these standards do not take into account trend accuracy, which is a potential advantage of CGM devices. In addition, the ISO and the FDA standards were created for intermittent monitoring, with only the consensus standard [10] developed for CGM.

Importantly, the results of the present study were obtained by comparing samples from different sites (arterial and venous), which may have affected the accuracy, and after filtering out the effects of several substances that interfered with the signal detected by the OptiScanner®. As a result of the adjustment of the algorithm for new interferents, the “no-read” rate was reduced. It is important that glucose monitoring systems can be adjusted for chemical confounders or interferents, and the finding of new interferents and subsequent need for the company to process data retrospectively highlights the importance of testing this device in patients receiving different treatments in different institutions.

Interestingly, glycemic control, as assessed by the TIR, was slightly lower with the CGM device. However, the difference in TIR between groups, although statistically significant, was probably not clinically relevant. Moreover, the TIR may be greater when CGM is used to guide an insulin treatment protocol that targets a range narrower than 80–150 mg/dl. Importantly, some episodes of hypoglycemia may have been missed by intermittent checks but were detected by the OptiScanner®, as shown in the example in Fig. 1c. However, there were no episodes of severe hypoglycemia, so we are unable to comment on the accuracy of the OptiScanner® in this setting. Nevertheless, the ability to detect hypoglycemia is an important safety issue, because even short episodes of iatrogenic or spontaneous hypoglycemia are associated with increased mortality [26]. Glucose variability indices appeared to be differentially affected by the time intervals between readings. The present data suggest that J-index, SD and MGC are accurate regardless of the measurement frequency and that GVI and GLI are the most time-influenced indices. Correct interpretation of indices of glucose variability thus requires knowledge of the device used to measure the blood glucose values.

The CGM device assessed in this study compares well with intravascular sensors that use other techniques, including enzymatic reactions [27], fluorescence [28, 29] and microdialysis [30]. One advantage of the OptiScanner® is that it does not require placement of an additional central catheter, in contrast to other systems that require peripheral venous access [27] or dedicated arterial access [29].

This study has some limitations, including lack of calculation of the impact of the CGM-based system on nursing workload or cost-effectiveness and the fact that we were unable to evaluate the accuracy of the CGM device in the severe hypoglycemic range. Furthermore, we had some practical problems with the device, as reflected by the downtime. Apart from the no reads, the most common difficulty was occlusion (blood draw and blood return) followed by the presence of bubbles. Once identified, the problem was resolved in most cases by flushing the system or manipulating the catheter. Nevertheless, because of these interventions and problems related to the cartridge, we sometimes had to prematurely disconnect the device.

## Conclusion

Based on the MARD, the performance of the OptiScanner® is adequate for use in ICU patients, even though other standards, initially designed for point-of-care glucose monitoring devices or suggested for CGM, were not met. Although problems related to the blood draw system need to be resolved, this device may represent an important, minimally invasive means of glucose monitoring in ICU patients. The assessment of glycemic variability is influenced by the time interval between BG determinations.

## Abbreviations

CEG: Clarke error grid
CGM: Continuous glucose monitoring
CV: Coefficient of variation
GLI: Glucose lability index
GVI: Glucose variability index
ICU: Intensive care unit
MAGE: Mean amplitude of glycemic excursions
MARD: Mean absolute relative difference
MGC: Maximal glucose change
PCV: Population coefficient of variation
SD: Standard deviation
TGC: Tight glycemic control
TIR: Time in range
